# Treatment of Invasive Aspergillosis: How It’s Going, Where It’s Heading

**DOI:** 10.1007/s11046-023-00727-z

**Published:** 2023-04-26

**Authors:** Johannes Boyer, Simon Feys, Isabella Zsifkovits, Martin Hoenigl, Matthias Egger

**Affiliations:** 1 Division of Infectious Diseases, Department of Internal Medicine, ECMM Excellence Center for Medical Mycology, Medical University of Graz, Auenbruggerplatz 15, 8036 Graz, Austria; 2 Medical Intensive Care Unit, University Hospitals Leuven, Louvain, Belgium; 3 Department of Microbiology, Immunology and Transplantation, KU Leuven, Louvain, Belgium; 4 BioTechMed, Graz, Austria

**Keywords:** Invasive aspergillosis, Therapy, Immunotherapy

## Abstract

Despite improvements in treatment and diagnostics over the last two decades, invasive aspergillosis (IA) remains a devastating fungal disease. The number of immunocompromised patients and hence vulnerable hosts increases, which is paralleled by the emergence of a rise in IA cases. Increased frequencies of azole-resistant strains are reported from six continents, presenting a new challenge for the therapeutic management. Treatment options for IA currently consist of three classes of antifungals (azoles, polyenes, echinocandins) with distinctive advantages and shortcomings. Especially in settings of difficult to treat IA, comprising drug tolerance/resistance, limiting drug–drug interactions, and/or severe underlying organ dysfunction, novel approaches are urgently needed. Promising new drugs for the treatment of IA are in late-stage clinical development, including olorofim (a dihydroorotate dehydrogenase inhibitor), fosmanogepix (a Gwt1 enzyme inhibitor), ibrexafungerp (a triterpenoid), opelconazole (an azole optimized for inhalation) and rezafungin (an echinocandin with long half-life time). Further, new insights in the pathophysiology of IA yielding immunotherapy as a potential add-on therapy. Current investigations show encouraging results, so far mostly in preclinical settings. In this review we discuss current treatment strategies, give an outlook on possible new pharmaceutical therapeutic options, and, lastly, provide an overview of the ongoing research in immunotherapy for IA.

## Introduction

Invasive aspergillosis (IA) caused by *Aspergillus* species, remains the predominant invasive mold infection [1–3]. These fungi cause life-threatening diseases mainly affecting immunocompromised patients with underlying hematological disease, cancer, autoimmune diseases, as well as solid organ transplant (SOT) recipients or critically ill patients, including those with respiratory viral infections [4, 5]. The increasing number of patients at risk is accompanied by rising frequencies of fungal infections due to *Aspergillus* spp, as well as the more frequent affection of male patients [6–9]. Prevalence can significantly vary among geographic regions, different centers and patient populations [10–12]. Despite incomplete data on a global scale, reports of increasing rates of azole-resistant *Aspergillus* strains emerge, with Europe being central amongst others [13–21]. For example, a multicentric study from the Kyoto and Shyga region in Japan found 12.7% of studied *Aspergillus fumigatus* isolates to be azole-resistant [22]. A recently published report of the world health organization (WHO) on fungal pathogens recognizes invasive fungal diseases as a rising global health concern, with *Aspergillus fumigatus* being included into the highest critical priority group [23]. *Aspergillus spp.* can infect many different sites of the body, but mainly involves the respiratory tract through inhalation of conidia [24]. Therapeutic measures for IA currently consist of three antifungal drug classes, namely azoles (voriconazole, posaconazole, isavuconazole), polyenes [liposomal amphotericin-B (LAmB), amphotericin-B lipid complex (ABLC)] and echinocandins (caspofungin, anidulafungin, micafungin) [25–28]. The emergence of azole-resistance, drug–drug interactions, and toxicity, is often limiting current therapeutic approaches [12, 29–31]. The future looks brighter however, with a number of new antifungals in development. Some of these introduce novel mechanisms of action, and all show promising results is first clinical studies, as well as excellent in vitro activity against most human pathogenic *Aspergillus* spp. [32, 33]. In this review we briefly discuss the current therapy standards of IA, primarily focusing on its pulmonary manifestation. Further, we want to present the novel antifungal agents regarding their potential future use in the treatment of IA and, lastly, we want to give insights into the current development stage of immunomodulatory therapy for IA.

### How it’s going

For the current treatment of IA, three classes of antifungal agents are available: Azoles, polyenes and echinocandins [34]. When IA is suspected, antifungal treatment needs to be initiated as early as possible, since delayed initiation of appropriate therapy is associated with worse outcomes [35, 36]. Pharmacokinetics and dosing regimens of current antifungal are displayed in Table 1.

**Table 1:** Pharmacokinetics of current and future drugs for the treatment of invasive Aspergillosis (derived in part from [37])

	Current first line agents		Future options				
	Voriconazole	Isavuconazole	Olorofim	Fosmanogepix°	Ibrexafungerp	Opelconazole°	Rezafungin
Intravenous standard dose	Loading dose 6 mg/kg b.i.d. on day1 Then 4 mg/kg b.i.d	Loading dose 200 mg t.i.d. on day 1 and day 2 Then 200 mg once daily	Early development	1000 mg IV b.i.d. on day 1 then 600 mg IV q.i.d. for at least 2 days, followed by 600 mg IV qd or 700 mg PO qd	IV formulation under development	14.8 mg nebulized b.i.d	400 mg IV in week 1, then 200 mg IV once weekly
Oral standard dose	Loading dose 400 mg b.i.d. on day1 Maintenance dose 200 mg b.i.d	Loading dose 200 mg t.i.d. on day 1 and day 2 Maintenance dose 200 mg once daily	150 mg b.i.d. on day1 Then 90 (− 150 mg) b.i.d	700 mg PO qd	750 mg b.i.d. for 2 days then 750 mg PO qd Combination with azoles: 500 mg PO b.i.d. for 2 days then 500 mg PO qd		–
Oral bioavailability	> 90%	98%	45%	> 90%	35–51%	–	–
*C*_max_ [µg/mL]	4.4 after i.v. administration	2.6	2.2	2.02	0.9	0.001	22.7
AUC [µg·h/mL]	30 after i.v. administration	34	11.9	4.49	26	0.017	
*V*_d_ [L/kg]	4.5	~ 6.5	3	/	600	> 5	89
Protein binding [%]	58	98–99	99.7	99.8	99	97	97
*t*_1/2_ [h]	~ 6	80–120	24–30	60	20–30	132	81
Metabolism and elimination	Hepatic metabolism involving 2C9, 2C19, and CYP3A4	Hepatic metabolism involving UGT, and CYP3A4	Hepatic metabolism	Manogepix active form Biliary excretion	Hydroxylation by CYP3A4 Biliary excretion	Hepatic metabolism	Hepatic metabolism 70% feces, 30% urine
Renal impairment	Standard dose, consider SBECD accumulation during i.v. infusion	Standard dose	–	–	–	–	Renal clearance < 1%
Hepatic impairment	Mild to moderate: 50% dose reduction, TDM recommended	Mild to moderate, enhanced levels, no dose reduction recommended by the manufacturer	–	–	–	–	–
Remark	Strong inhibitor of CYP2C9 and 2C19, moderate inhibitor of CYP3A4	Inhibitor of CYP3A4, P-gp and BCRP	Weak inhibitor CYP3A4	Interaction profile not yet known	Inhibitor of CYP3A4 and CYP2C8 Low brain/eye c.n.c	CYP3A4/CYP2C9 substrate	Low potential for drug interactions
PK/PD target	AUC_0–24h_/MIC	AUC_0–24h_/MIC	Cmin/MIC	AUC_0–24h_/MEC	AUC_0–24h_/MEC (vs. *Aspergillus)*	–	AUC_0–24h_/MEC and Cmax/MEC (vs. *Aspergillus)*
REF			[38]	[38, 39]	[38, 40]	[41]	[38, 42]

°PK parameters following repeated 5 mg dosing

*C*_max_, peak level; i.v. intravenous; AUC, area under the concentration time curve; *V*_d,_ apparent volume of distribution; *t*_1/2_ half-life; CL clearance; GFR, glomerular filtration rate; CYP cytochrome P 450; SBECD, sulfobutylether-*β*-cyclodextrin; TDM, therapeutic drug monitoring; UGT, uridine diphosphate glucuronosyltransferase, P-gp, P glycoprotein, and BCRP, breast cancer related protein. AUC_0–24 h_, area under the concentration–time curve over 24 h; MIC, minimal inhibitory concentration of the *Aspergillus* strain; c.n.c., concentration; qd, once daily; b.i.d., twice daily; t.i.d., three times daily; q.i.d., four times daily

#### Azoles

For nearly two decades, voriconazole is recommended as first-line therapy for IA [34, 35, 43]. It has shown superior results when compared to amphotericin-B deoxycholate (D-AmB) [34, 44]. In the most significant studies concerning treatment of IA, voriconazole showed about 30% overall-mortality on day 84, with reports of response rates ranging between 36% and 52.8% [44–47]. The initial use of voriconazole was associated with decreased length of hospital stay in a sub-group analysis of the TRANSNET-population [48]. In cases of disseminated diseases, it is an important agent known for its good central nervous system (CNS) and eye penetration [49, 50]. The most common side effects of voriconazole include hepatotoxicity, temporary visual disorders, phototoxic phenomena (e.g. skin rash, erythroderma), perioral excoriations, tachyarrhythmias, and psychiatric side effects such as hallucinations (often due to iv administration; reversible; may disappear during treatment or switch to oral formulation) with some of them being dose dependent [45, 51–53]. Due to marked drug–drug interactions [54, 55] and pharmacokinetic variability in absorption, distribution, metabolism and clearance of antifungals, therapeutic drug monitoring (TDM) is an important tool to ensure adequate therapeutic levels [45, 54, 56]. Plasma levels of voriconazole should be controlled 2–5 days after the first dose. If levels are sufficient (between 1–1.5 and 5–6 μg/mL), they should be monitored regularly due to high intraindividual variation. At a minimum, the next measurement should be performed when a change in the patient’s condition or co-medication is observed (e.g, clinical alterations, new concurrent medication, presumed toxicity) [54, 57].

Isavuconazole is an alternative first-line agent with high tolerability and fewer side effects [34, 58, 59]. ECIL-6 guidelines state that isavuconazole is equally effective as voriconazole [35, 55]. For the treatment of mold disease, isavuconazole was non-inferior compared to voriconazole, with some benefits especially regarding pharmacokinetics, where it has shown superior accessibility and more linear pharmacokinetic properties [45]. Even in the context of limited data for target drug levels and much less variation in levels compared to voriconazole, TDM is still recommended in special clinical settings, like patients on extracorporeal membrane oxygenation (ECMO) or continuous renal replacement therapy [54, 58, 60]. Proposed thresholds vary between > 1–2.5 µg/ml for the lower limit and 4–5 µg/ml for the upper limit. Both aim to improve efficacy and safety of isavuconazole, yet further validation is needed [61–65].

In case of intolerance or refractory disease after 7 days of therapy with a first-line antifungal, a switch of drug class, meaning a switch from an azole to LAmB in most cases, is recommended [66, 67]. Initiation of combination therapy with e.g. an echinocandin being an alternative. In some cases with adverse events or insufficient voriconazole drug levels, also isavuconazole or posaconazole in tablet/intravenous formulation are potential alternatives [34, 54].

Regarding posaconazole, a randomized-controlled non-inferiority trial published in 2021 showed non-inferiority to voriconazole with respect to all-cause mortality, and adverse effects were less common [44]. Tablet and intravenous formulations should be used preferentially over posaconazole suspension formulations, achieving more reliable therapeutic doses [68–71]. Nevertheless, plasma concentrations of posaconazole should also be monitored, preferably on day 5 after the initiation of treatment [54, 70, 72]. Suggested plasma levels are > 0.7 µg/ml for prophylaxis and > 1 µg/ml for treatment with an upper threshold of 3.75 µg/ml [28, 73].

Indications for itraconazole are very limited, due to its poor absorption an unpredictable pharmacokinetics, but can be considered as an alternative agent in settings with restricted resources [34].

Generally, azoles can cause various drug–drug interactions. Notably, these include interactions with commonly used immuno suppressive agents such as tacrolimus, sirolimus and cyclosporine, potentially resulting in pronounced immunosuppression or toxicity [74]. Under such circumstances dose adjustment is essential. Other potential drug–drug interactions may occur together with targeted hematological therapies, antiretroviral agents, and, amongst many others, anticoagulants [75–77].

#### Polyenes

Lipid formulations of amphotericin-B such as LAmB and ABLC are recommended for second line or salvage therapy of IA. These formulations are associated with decreased levels of nephrotoxicity and are preferable in settings of liver impairment, with LAmB being considered the drug of choice since it is better tolerated than ABLC [78–80]. Common side effects include flush, infusion-related events (e.g. fever, chills), nephrotoxicity and hypokalemia [81]. For LAmB, nephrotoxicity can be expected in about 10–25% and hypokalemia in about 15%. As a consequence, routine monitoring of electrolytes and renal function is advised [81–83]. When azoles cannot be administered due to contraindications or intolerabilities, LAmB is a viable alternative [35, 82]. The AmBiLoad-Trial demonstrated that, for the treatment of IA, higher doses of LAmB (10 mg/kg per day) have no additional benefits and are associated with higher toxicity compared to standard doses (3 mg/kg per day) [35, 82]. A major benefit of polyenes is that acquired antifungal resistance has not been reported despite the drug now being used for many decades. D-AmB is no longer endorsed, due to multiple associated adverse effects, especially renal toxicity [44, 55, 78]. In exceptional situations however, it can be used when no other antifungal drugs are available [35]. D-AmB and lipid formulations are—off label—sometimes also utilized as aerosolized formulations in combination with systemic antifungal therapy [34], as well as for prophylaxis in cases of prolonged neutropenia or in lung transplant recipients [35, 84–86]. High drug concentrations in the airways and less systemic side effects have been reported as noticeable advantages of combined therapy [84].

#### Echinocandins

The echinocandins (caspofungin, anidulafungin, micafungin) are rarely used as monotherapy due to limited clinical efficacy, and therefore strongly recommended only in combination with other antifungals [34, 35]. An expert opinion published in 2015 on azole-resistant strains of *Aspergillus fumigatus* favor LAmB over echinocandin-monotherapy [87]. Caspofungin is known as the only echinocandin which is approved by the FDA to treat IA in the setting of salvage therapy [34, 88–90]. While effective against *Aspergillus* spp. in vitro, there is insufficient clinical data in the treatment of IA regarding micafungin and anidulafungin as monotherapy [91, 92].

#### Combination therapy

As first-line therapy, the combination of antifungal agents is not primarily recommended [54]. In salvage therapy however, the usage of an echinocandin together with an azole or with LAmB can be taken into consideration [34, 35, 54]. In a randomized trial, the combination of voriconazole with anidulafungin was analyzed in contrast to voriconazole as monotherapy, especially regarding safety and efficacy concerns. The mortality rates after 6 weeks of treatment, as the primary outcome, and the mortality rates after 12 weeks of therapy, in addition to mortality in subgroups and safety concerns as secondary outcome, were investigated. Mortality rate after 6 weeks was 19.3% in the group that received combination therapy and 27.5% in the monotherapy group. No statistically significant differences were observed in terms of safety and toxicity [93].

As a result, combination therapy should be utilized in specific settings such as those with (1) high azole-resistance rates [94] (2) in cases with lacking response to monotherapy (3) when the species of *Aspergillus* is unknown or susceptibility testing is pending (4) or when therapeutic level of voriconazole cannot be reached due to poor metabolism by concurrently administered medication. For CNS infections, combination therapy may be complicated due to concerns about antagonism between voriconazole and LAmB and the poor brain and cerebro−spinal fluid penetration of echinocandins [34, 95]. The antimetabolite flucytosine is another option, particularly in combination with LAmB for severe *Cryptococcal* infections (meningitis, pneumonia), difficult to treat invasive *Candida* infections, as well as for urogenital infections involving fluconazole-resistant *C. glabrata* [96]. Flucytosine has high oral bioavailability and distributes widely into tissues including the cerebro-spinal fluid. Adverse events include liver enzyme elevation and dose dependent myelosuppression [97]. LAmB induced nephrotoxicity may lead to deacreased excretion and hence increased concentrations.

#### Duration

The duration of therapy should be primarily guided by clinical, microbiological, and radiographic response, with an absolute minimum treatment duration of 6 weeks, while most of the patients receive treatment for at least 12 weeks [34]. Duration is further linked to the degree and foreseeable length of immunosuppression, as well as the site of disease [35]. Secondary prophylaxis for immunosuppressed patients after recovery is also highly recommended for preventing recurrence of IA, especially if patients require further immunosuppressive therapy [35]. The optimal duration of therapy is commonly determined individually.

### Where it’s heading

#### New antifungals

Availability of only three antifungal drug classes for the treatment of IA is drastically restricting therapeutic options. Especially in settings of difficult to treat IA, comprising drug tolerance/resistance, limiting drug–drug interactions, and/or severe underlying organ dysfunction, agents with innovative mechanisms of action and beneficial pharmacokinetic properties are urgently needed. Current antifungals in the pipeline address these needs and will substantially extend and enhance treatment options for IA [32]. Pharmacokinetics of current first line agents compared to novel options are displayed in Table 1.

A representative of a new antifungal drug class (i.e. orotomide) is olorofim. It inhibits the dihydroorotate dehydrogenase, an enzyme involved in pyrimidine synthesis, and shows good activity against various *Aspergillus* spp. including azole-resistant strains and difficult to treat cryptic species [98]. The compound is highly protein-bound and shows excellent tissue distribution in lung, liver, kidney and brain. Although olorofim is metabolized by CYP450 enzymes and hence sensitive to CYP inhibitors/inducers, it appears to only have mild effects on CYP enzymes, which renders it an agent with low potential for drug–drug interactions [99]. It will be available as oral formulation and a promising option for IA monotherapy, particularly in settings of azole-resistant species. Most recent studies investigating the efficacy of olorofim in therapy of IA, include the OASIS-study (NCT05101187) where olorofim is compared with LAmB followed by standard of care in a Phase III, adjudicator-blinded, randomized study. Another phase IIb clinical trial investigating olorofim in invasive fungal diseases with limited treatment options including IA (NCT03583164) is currently in the final steps of its recruiting phase.

A further novel mechanism of action is introduced by fosmanogepix. By inhibiting Gwt1, an enzyme essential for anchoring mannoproteins to the fungal cell membrane and wall, fungi cannot adhere to mucosal and epithelial surfaces, which is prerequisite for colonization/infection. It has broad activity against *Aspergillus spp.* including azole-resistant strains and is developed as oral and IV formulation [100]. Further, in a phase II trial including 66 patients with renal insufficiency, administration of fosmanogepix neither resulted in worsening of renal function, nor was a dose adjustment required, outlining the potential safety in this setting, especially when adding fosmanogepix to LAmB [101], a combination which has shown strong synergism in animal models [100]. It could constitute a promising treatment option for IA as monotherapy or in combination with other classes when the disease is difficult to treat. The open-label AEGIS-study (NCT04240886) evaluating fosmanogepix in therapy of IA or rare mold infections was completed in May 2022 with results yet to be published.

The oral glucan synthase inhibitor ibrexafungerp has broad antifungal activity including azole-resistant and cryptic *Aspergillus* species [40, 102]. The mechanism of action is similar to that of echinocandins, yet the binding site differs slightly resulting in low cross-resistance. Its spectrum of activity together with favorable pharmacokinetic/pharmacodynamic properties (i.e. high tissue penetration, favorable drug–drug interaction and side effect profile) render it a valuable agent for resistant IA treatment and an alternative for oral (combination) step-down when azoles lack applicability. Furthermore, a multicenter, randomized, double-blind study investigating the safety and efficacy of the co-administration of voriconazole and ibrexafungerp in patients with invasive pulmonary aspergillosis (IPA) (NCT03672292) is in the recruitment phase. Also the FURI-study (NCT03059992), evaluating the efficacy and safety of ibrexafungerp in refractory fungal disease (including as a combination treatment component for IA) or in patients intolerant to standard therapy, is estimated to be completed in August 2023.

Opelconazole was particularly designed for inhalation therapy (through adapted particle size) and could be a most welcome add-on in settings where systemic administration is limited due to toxicity. Opelconazole shows broad activity against *Aspergillus* spp. and enables high local concentrations while avoiding systemic adverse effects. Synergism has been observed with systemically administered azoles, indicating suitability for a combination approach in difficult to treat IA [103]. A double-blind, randomized, placebo-controlled study assessing the efficacy and safety of opelconazole when added to systemic antifungal therapy in refractory IA (NCT05238116) is currently recruiting patients and is estimated to be completed in 2024 [32].

Lastly, rezafungin, a second-generation echinocandin with optimized pharmacokinetics (e.g. mean half-life of ~ 150 h after two dosages), will allow, amongst other options, outpatient therapy in combination with oral agents [42]. These could include combinations with fosmanogepix, olorofim, ibrexafungerp or conventional azoles, with data on potential synergistic/antagonistic effects needed.

**Fig. 1:**
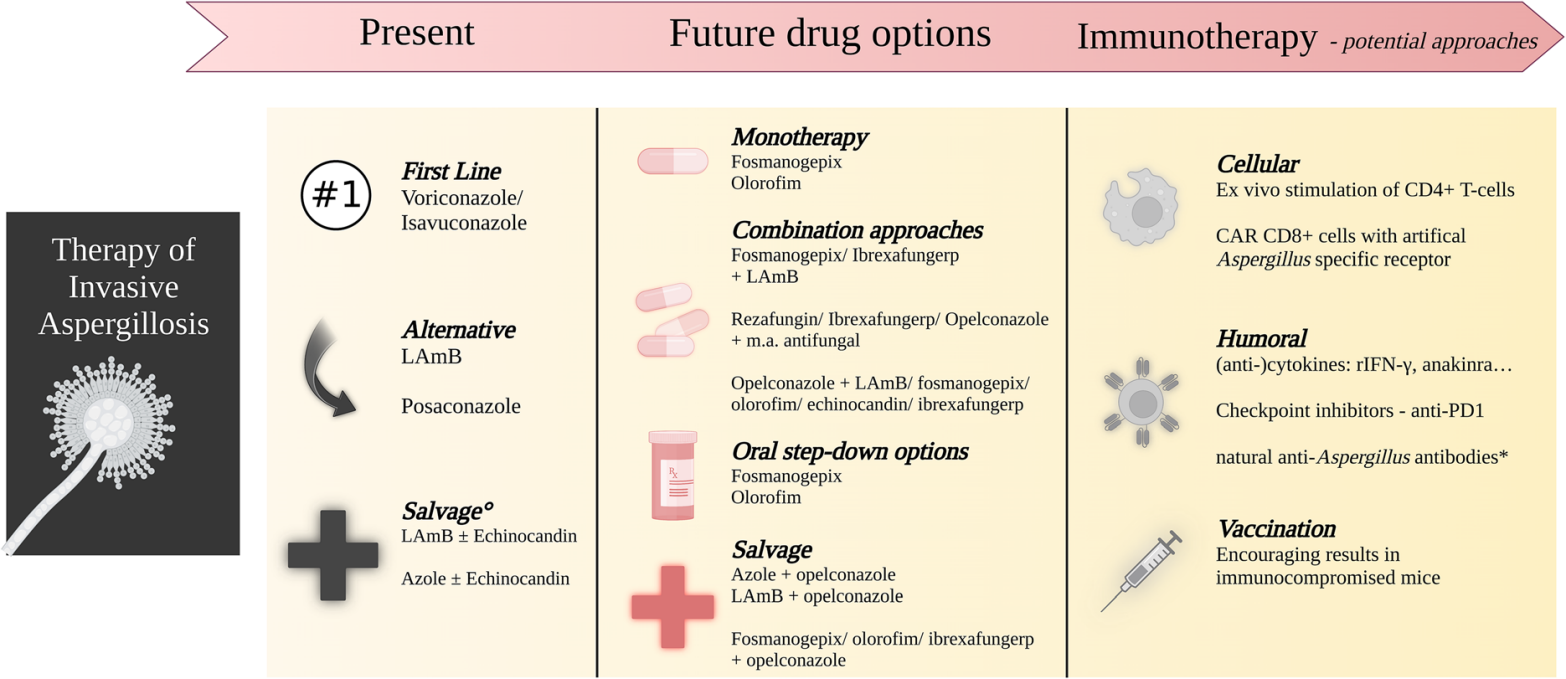
Therapy of invasive aspergillosis—current approach and outlook. LAmB = liposomal amphotericin-B; m.a. = mold active; CAR = chimeric antigen receptor; rIFN = recombinant infereron; PD-1 = programmed cell death protein 1. °In case of monotherapy, class need to be switched; Azole is an option in case of initial treatment with LAmB, insufficient plasma levels, or limiting drug–drug interactions/adverse events. *Beneficial results in mouse model of influenza and pseudo-COVID-associated pulmonary aspergillosis

Current and future treatment approaches are displayed in Fig. 1.

### Immunotherapy

Incremental insight into the pathophysiology of IA starts to create traction for immunotherapy as add-on treatment or prophylaxis. Immunotherapy does not target *Aspergillus* in a direct way like antifungals but boosts the antifungal host response to better clear spores and/or hyphae. Possible strategies to improve antifungal immunity involve humoral (i.e., antibodies, cytokines, cytokine-blockade, checkpoint-inhibitors) or cellular approaches and vaccination.

In preclinical work and in a limited number of case reports or case series in IA, beneficial results have been seen with recombinant interferon-gamma (rIFN-*γ*), which increases phagocytic antifungal activity, and checkpoint-inhibitors (e.g. anti-PD-1), which counter immune cell exhaustion [104–117]. The potential of rIFN-*γ* has been investigated more thoroughly in the setting of prevention of IA in patients with chronic granulomatous disease specifically, and in chronic pulmonary aspergillosis [118, 119]. Likewise, mouse models and case series showed potential for granulocyte-macrophage colony-stimulating factor (GM-CSF) in IA, but a randomized phase IV clinical trial could not show benefit for GM-CSF with or without granulocyte colony-stimulating factor (G-CSF) as prophylaxis for IA after allogeneic stem cell transplantation [109, 120–123]. Administration of other humoral innate immune agents such as pentraxin-3 (PTX3) or surfactant–protein D (SP-D) are protective in vitro and/or in mice, but their potential for treatment of IA has not been investigated yet in a clinical setting [124–127].

Allogeneic granulocyte transfusion is a conceptually interesting approach in neutropenic patients, but this technique needs further optimization as several trials were unable to show benefit regarding IA [128, 129]. T-cells currently show more promise for IA treatment. Adoptive T-cell transfer, in which a patient’s CD4 + T-cells are stimulated with *Aspergillus *ex vivo and then re-infused, was beneficial in allogeneic stem cell transplant recipients with IA [130]. The use of chimeric antigen receptor (CAR) CD8 + T-cells, expressing an artificial T-cell receptor specific to *Aspergillus*, may have even more potential and recently showed encouraging results in a mouse model [131].

Positive results have been obtained in several mouse models regarding vaccination to prevent IA [132–135]. The largest issue to overcome to make vaccination a viable strategy to prevent IA is the requirement of a sufficiently working group of B- and T-cells, which is a problem in many severely immunocompromised IA patients [136]. Encouraging results were recently obtained by several groups in immunocompromised mice [137–139], but more work is needed before translation to the clinic.

Given the hyperinflammatory environment in the lungs of patients with influenza- or COVID-19-associated pulmonary aspergillosis (IAPA or CAPA), immunotherapy results obtained in classically immunocompromised mice or EORTC/MSGERC host factor positive IA patients are not readily translatable to IAPA/CAPA patients. Interesting immunomodulatory targets deserving further investigation in IAPA and CAPA patients specifically are rIFN-γ (given that decreased interferon-gamma signaling has been identified in IAPA/CAPA patients compared to non-IAPA/CAPA severe influenza and COVID-19 patients), anakinra (anti-interleukin-1, given the hyperinflammation and probable defect in LC3-associated phagocytosis in these patients) and natural anti-*Aspergillus* antibodies (shown to be decreased in patients with severe influenza or COVID-19 and proven beneficial in a IAPA mouse model) [140–142].

Due to the lack of high-quality trials in patients with IA, none of the approaches discussed above are currently part of standard of care for IPA (except for rIFN-*γ* prophylaxis patients with chronic granulomatous disease) [35]. High-quality clinical trials with the most promising candidates are therefore urgently needed to enable regular evidence-based use of immunomodulation as add-on therapy in IA.

## Conclusions

IA remains associated with high mortality despite improvement in therapy over the last two decades. Pharmaceutical options are currently limited with need of alternative agents, especially in the setting of salvage therapy or when azole-resistant strains are identified. Patients with IA are often multimorbid including impaired organ function limiting the use of azoles when hepatic impairment is present or the unrestrained use of LAmB in case of impaired kidney function. Further, azoles cause substantial interaction with important immunosuppressive agents such as tacrolimus, sirolimus and cyclosporine which can render management difficult and potentially result in pronounced immunosuppression or toxicity [74]. Another shortcoming of the current antifungal armamentarium is that the azoles present the only oral option for IA. While the optimal therapy duration is unknown, the Infectious Diseases Society of America recommends a minimum of 6 to 12 weeks therapy [35], which leads to organizational challenges (e.g. is a central line needed?, does the patient remain hospitalized only for IV therapy?, is an ambulant IV therapy practically possible?).

The new antifungals have the potential to complement the existing antifungal repertoire, and thereby improve patient outcomes. All novel agents appear to have an advantageous safety profile except for the need to adjust olorofim doseage when administered with strong CYP3A4 inhibitors or inducers, as well as potential opelconazole interaction with CYP3A4/5 CYP450-subenzyme, which is likely not relevant as the drug does not get absorbed from the lungs, no relevant drug–drug interactions have been observed. These circumstances nominate the new antifungals attractive options especially for SOT recipients. Furthermore, ibrexafungerp, fosmanogepix, opelconazole and olorofim can all be administered orally/via inhalation, making them suitable alternatives to azoles in the outpatient setting. Likewise, rezafungin with a once-weekly administration seems a viable option for these settings [32]. The clinical efficacy of novel antifungals for the treatment of IA still needs to be demonstrated, but first results look promising that the antifungal pipeline will provide the tools for improving the management of aspergillosis and associated upcoming challenges.

Regarding immunotherapy for IA, encouraging results have been obtained with different forms of immunotherapy in preclinical models and clinical trials including patients with pending. Different immunological backgrounds of patients at risk for aspergillosis (ranging from pronounced neutropenia to extreme hyperinflammation) will necessitate proper patient stratification to ensure tolerability and efficacy for each immunotherapeutic modality. Moreover, diagnostic immunological read-outs (e.g. measuring blood cytokines) might aid with identifying patients who could benefit from a selected immunotherapeutic. With this in mind, high-quality clinical trials in well-defined patient groups might lead to implementation of immunomodulatory prophylaxis or treatment for IA during the next decade.

To conclude, antifungal treatment of aspergillosis will likely substantially change over the next years, with new antifungals filling important gaps we are facing with current treatment options. Ultimately, the hope is that these changes will translate to better patient outcomes and survival.
